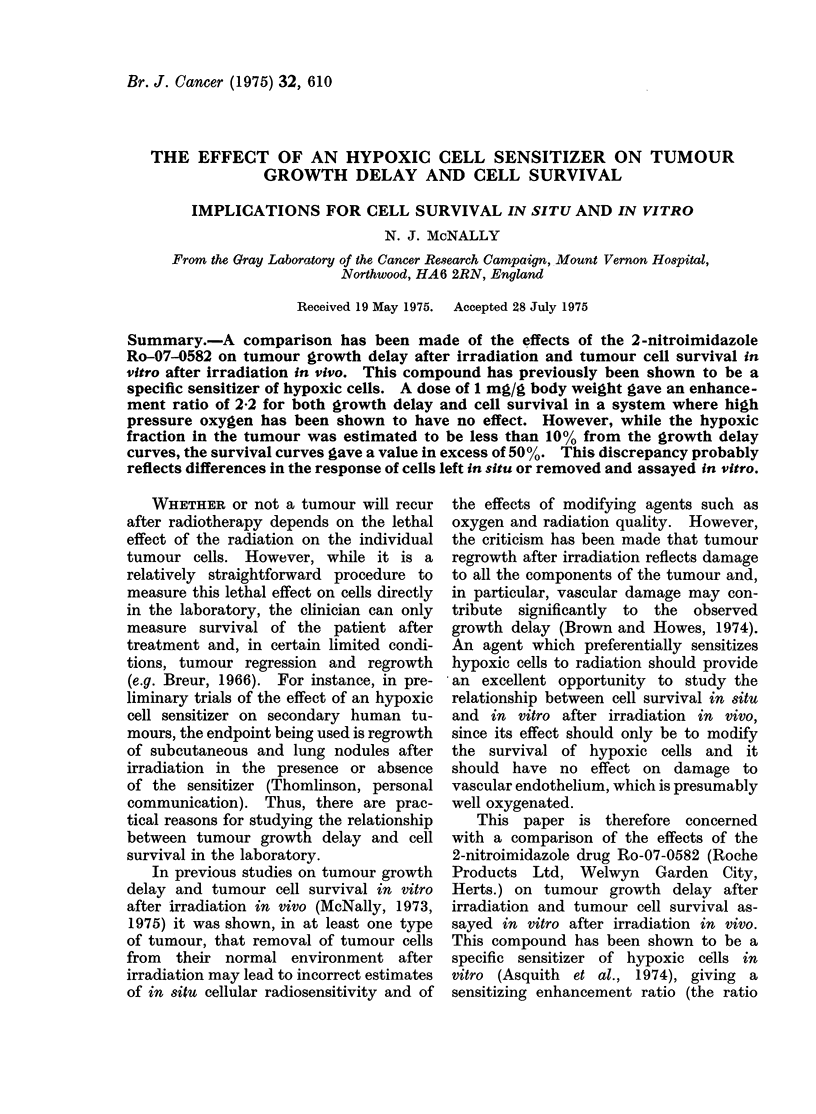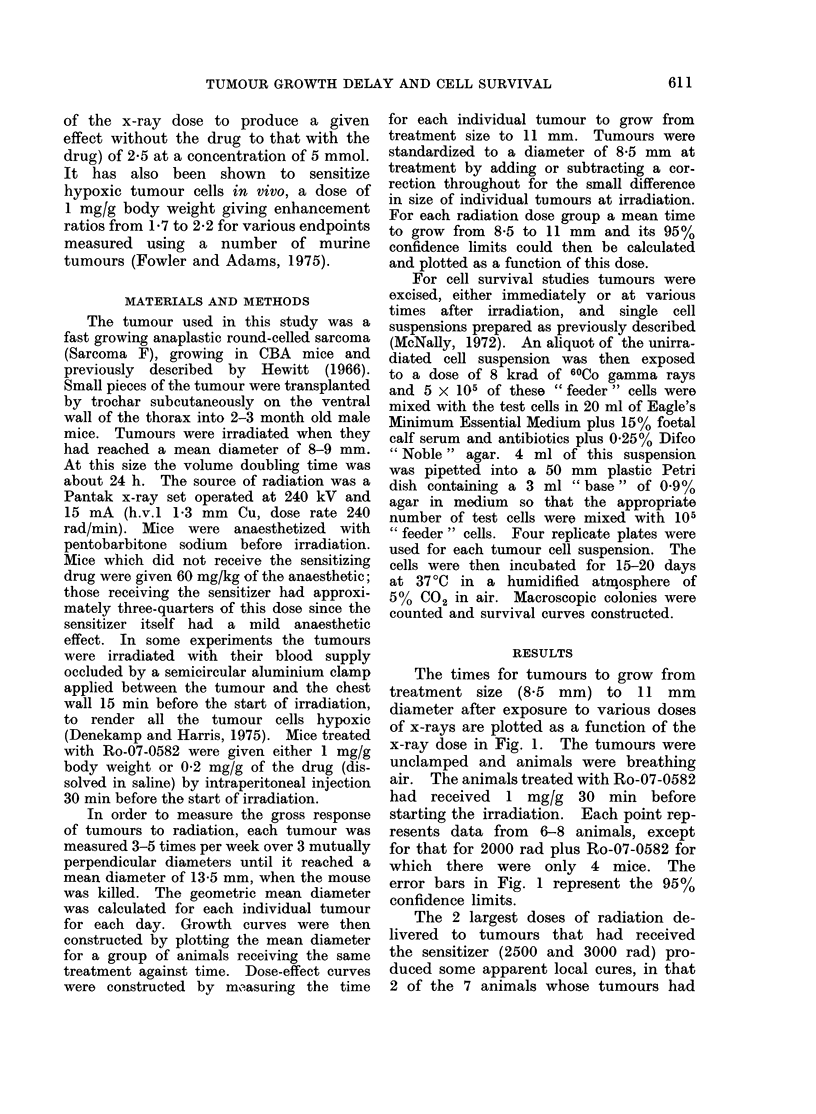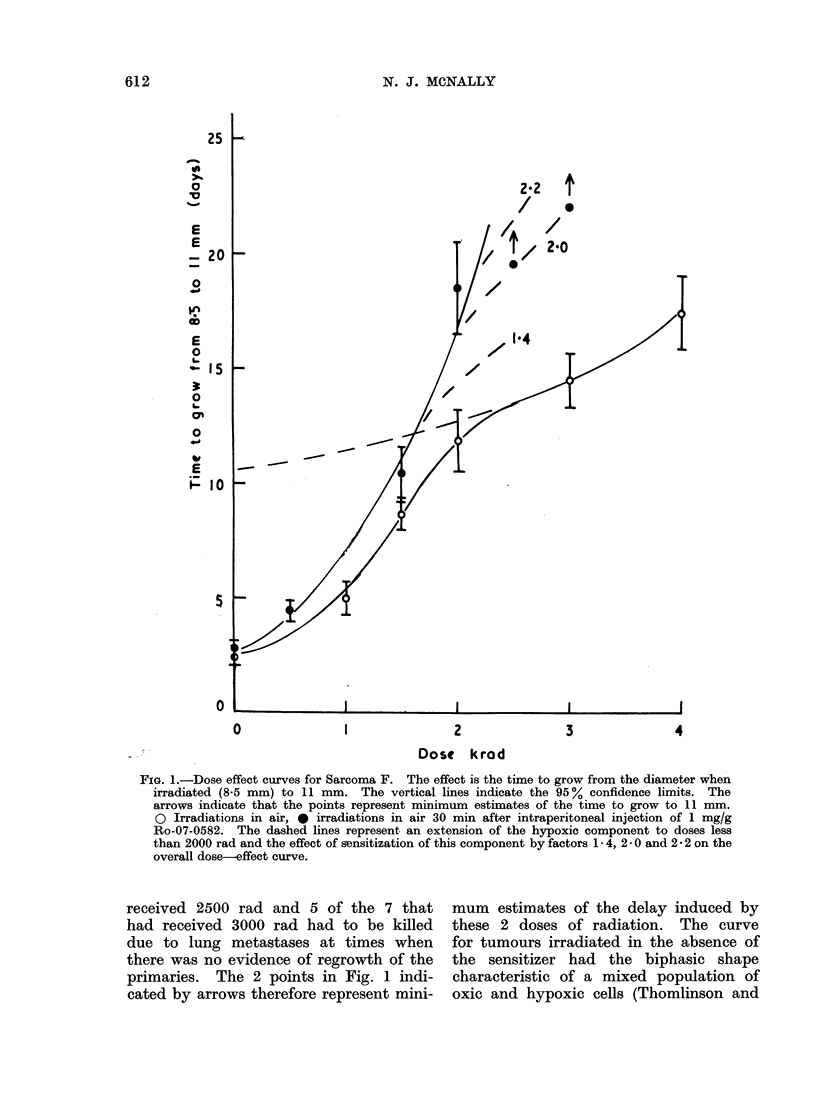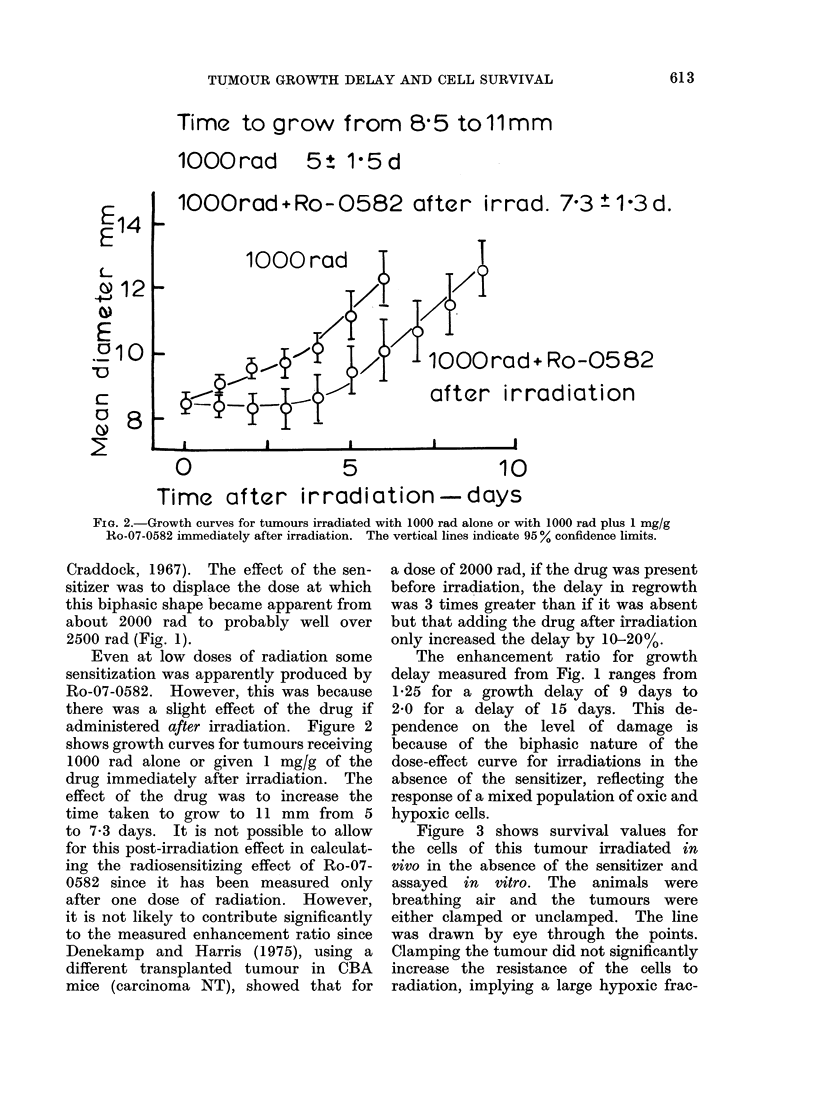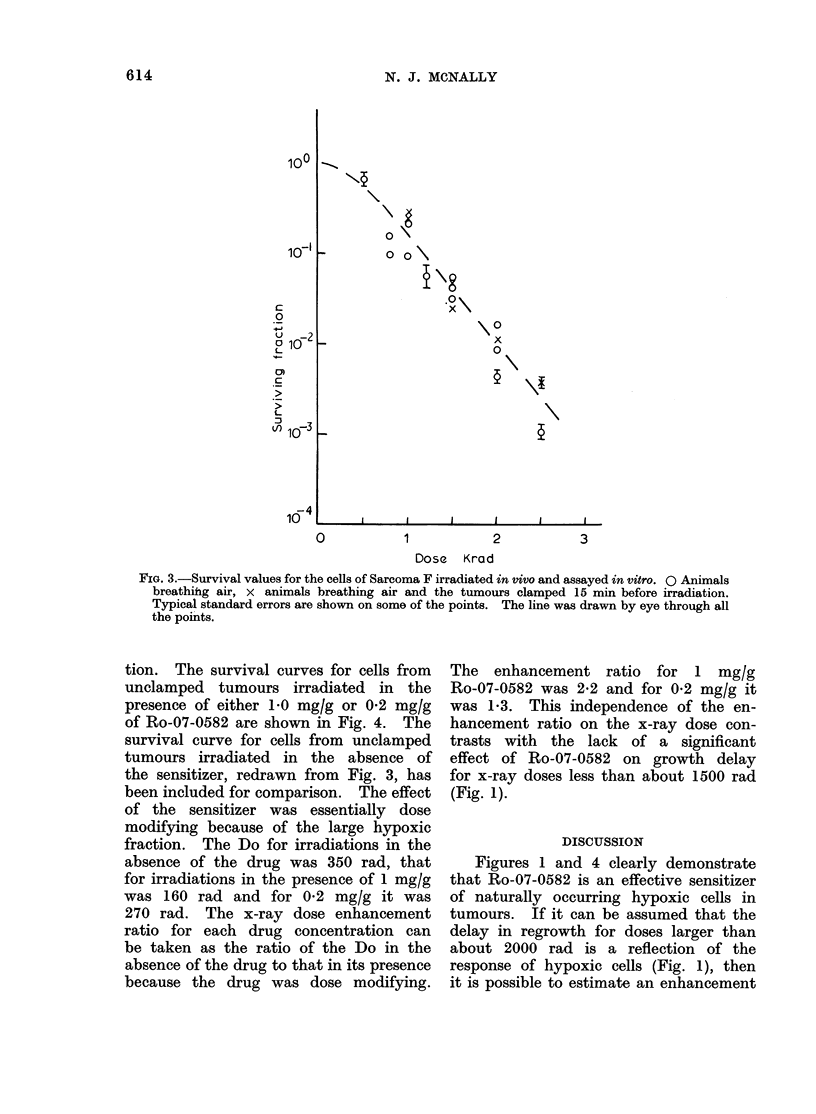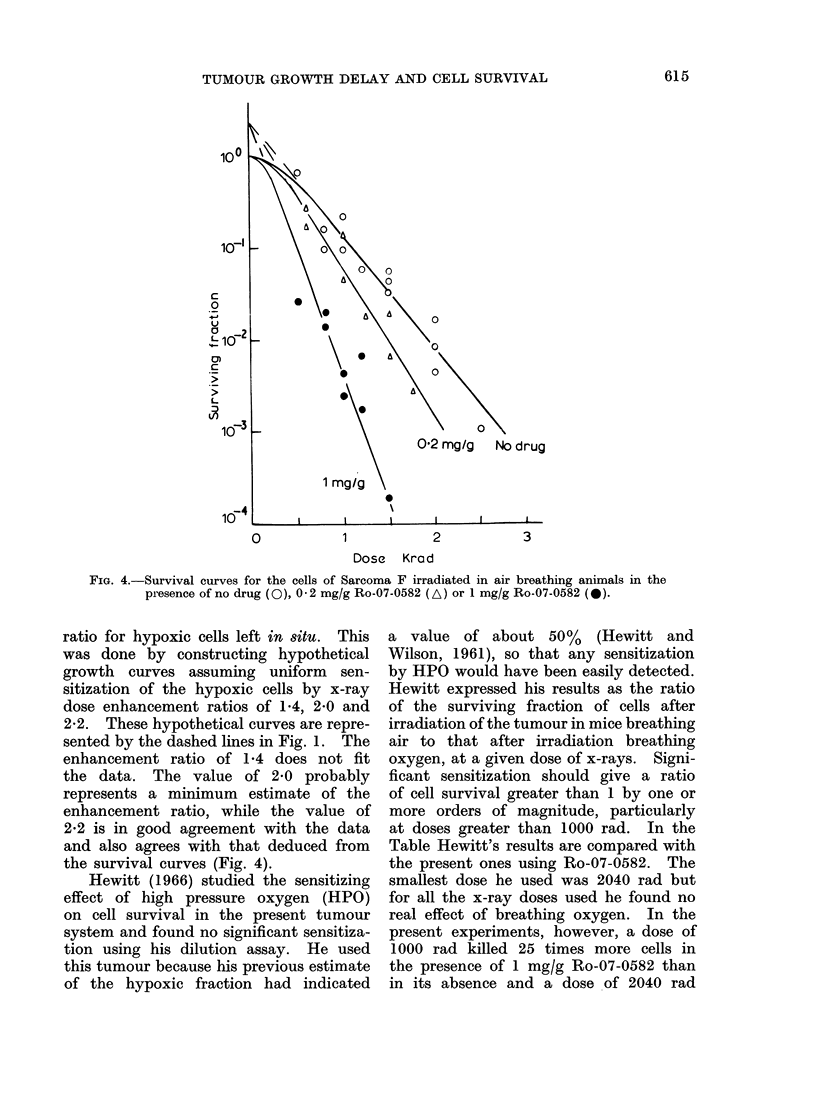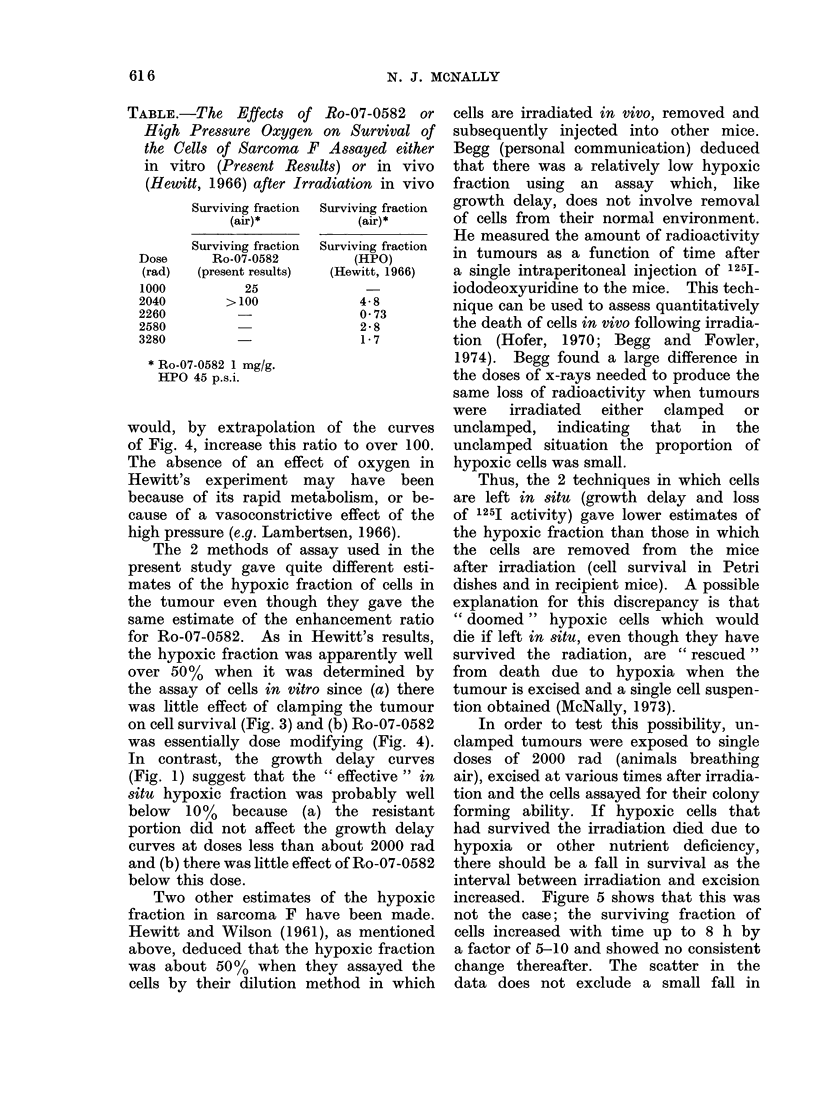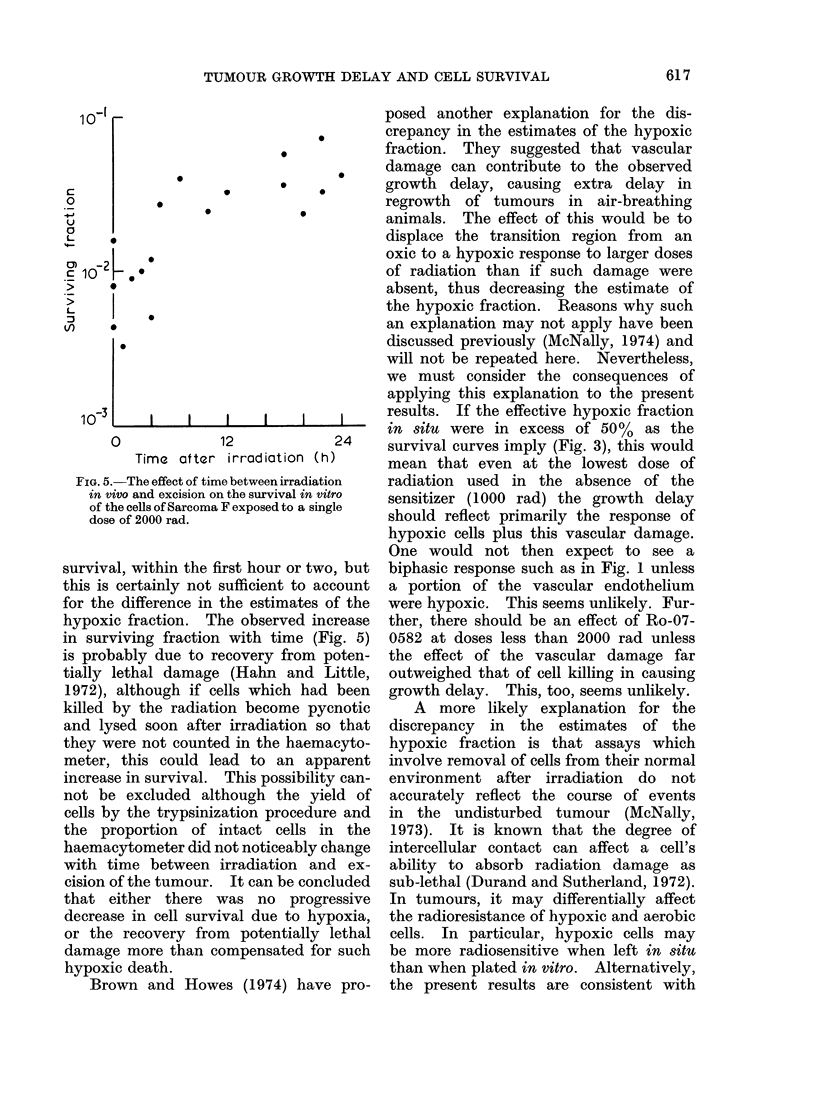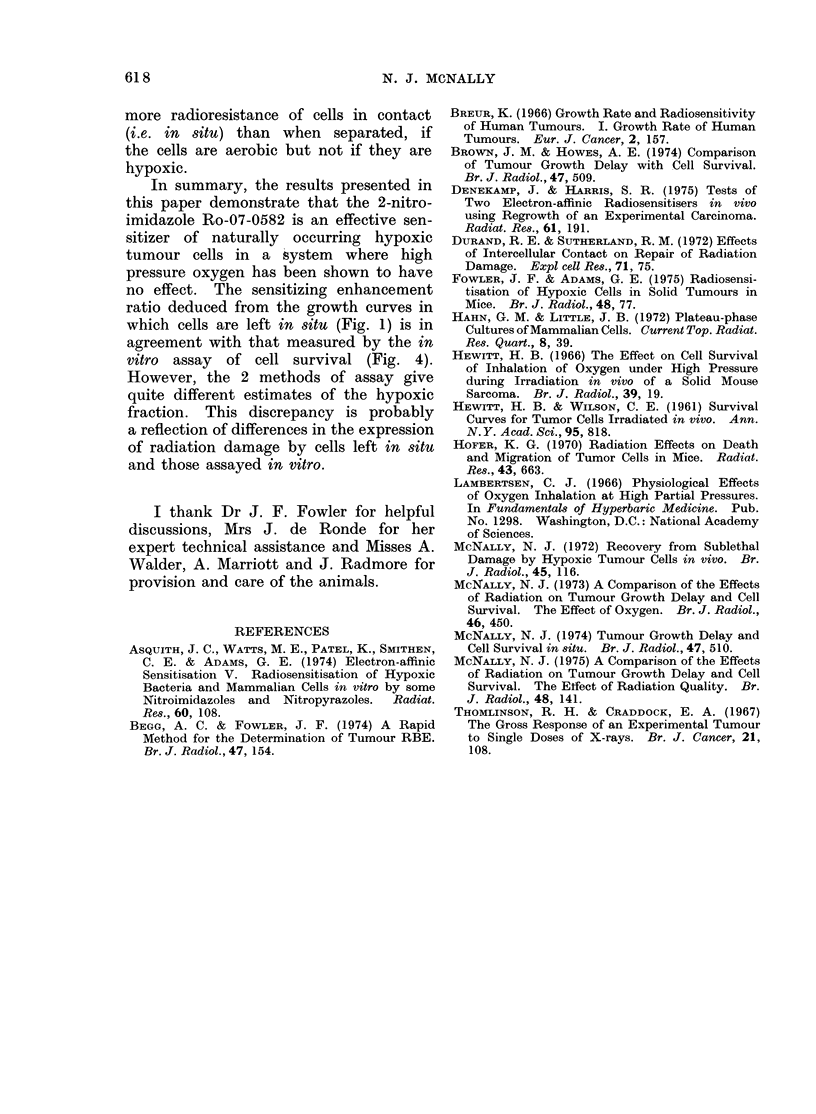# The effect of an hypoxic cell sensitizer on tumour growth delay and cell survival. Implications for cell survival in situ and in vitro.

**DOI:** 10.1038/bjc.1975.268

**Published:** 1975-11

**Authors:** N. J. McNally

## Abstract

A comparison has been made of the effects of the 2-nitroimidazole Ro-07-0582 on tumour growth delay after irradiation and tumour cell survival in vitro after irradiation in vivo. This compound has previously been shown to be a specific sensitizer of hypoxic cells. A dose of 1 mg/g body weight gave an enhancement ratio of 2-2 for both growth delay and cell survival in a system where high pressure oxygen has been shown to have no effect. However, while the hypoxic fraction in the tumour was estimated to be less then 10% from the growth delay curves, the survival curves gave a value in excess of 50%. This discrepancy probably reflects differences in the response of cells left in situ or removed and assayed in vitro.


*Br. J. Cancer* (1975) **32**, 610

### THE EFFECT OF AN HYPOXIC CELL SENSITIZER ON TUMOUR GROWTH DELAY AND CELL SURVIVAL

### IMPLICATIONS FOR CELL SURVIVAL *IN SITU* AND *IN VITRO*

N. J. McNALLY

*From the Gray Laboratory of the Cancer Research Campaign, Mount Vernon Hospital, Northwood, HA6 2RN, England*

Received 19 May 1975. Accepted 28 July 1975

**Summary.—A comparison has been made of the effects of the 2-nitroimidazole Ro–07–0582 on tumour growth delay after irradiation and tumour cell survival *in vitro* after irradiation *in vivo*. This compound has previously been shown to be a specific sensitizer of hypoxic cells. A dose of 1 mg/g body weight gave an enhancement ratio of 2·2 for both growth delay and cell survival in a system where high pressure oxygen has been shown to have no effect. However, while the hypoxic fraction in the tumour was estimated to be less than 10% from the growth delay curves, the survival curves gave a value in excess of 50%. This discrepancy probably reflects differences in the response of cells left *in situ* or removed and assayed *in vitro*.**

WHETHER or not a tumour will recur after radiotherapy depends on the lethal effect of the radiation on the individual tumour cells. However, while it is a relatively straightforward procedure to measure this lethal effect on cells directly in the laboratory, the clinician can only measure survival of the patient after treatment and, in certain limited conditions, tumour regression and regrowth (*e.g.* Breur, 1966). For instance, in preliminary trials of the effect of an hypoxic cell sensitizer on secondary human tumours, the endpoint being used is regrowth of subcutaneous and lung nodules after irradiation in the presence or absence of the sensitizer (Thomlinson, personal communication). Thus, there are practical reasons for studying the relationship between tumour growth delay and cell survival in the laboratory.

In previous studies on tumour growth delay and tumour cell survival *in vitro* after irradiation *in vivo* (McNally, 1973, 1975) it was shown, in at least one type of tumour, that removal of tumour cells from their normal environment after irradiation may lead to incorrect estimates of *in situ* cellular radiosensitivity and of the effects of modifying agents such as oxygen and radiation quality. However, the criticism has been made that tumour regrowth after irradiation reflects damage to all the components of the tumour and, in particular, vascular damage may contribute significantly to the observed growth delay (Brown and Howes, 1974). An agent which preferentially sensitizes hypoxic cells to radiation should provide an excellent opportunity to study the relationship between cell survival *in situ* and *in vitro* after irradiation *in vivo*, since its effect should only be to modify the survival of hypoxic cells and it should have no effect on damage to vascular endothelium, which is presumably well oxygenated.

This paper is therefore concerned with a comparison of the effects of the 2-nitroimidazole drug Ro-07-0582 (Roche Products Ltd, Welwyn Garden City, Herts.) on tumour growth delay after irradiation and tumour cell survival assayed *in vitro* after irradiation *in vivo*. This compound has been shown to be a specific sensitizer of hypoxic cells *in vitro* (Asquith *et al.*, 1974), giving a sensitizing enhancement ratio (the ratio of the x-ray dose to produce a given effect without the drug to that with the drug) of 2·5 at a concentration of 5 mmol. It has also been shown to sensitize hypoxic tumour cells *in vivo*, a dose of 1 mg/g body weight giving enhancement ratios from 1·7 to 2·2 for various endpoints measured using a number of murine tumours (Fowler and Adams, 1975).

### MATERIALS AND METHODS

The tumour used in this study was a fast growing anaplastic round-celled sarcoma (Sarcoma F), growing in CBA mice and previously described by Hewitt (1966). Small pieces of the tumour were transplanted by trochar subcutaneously on the ventral wall of the thorax into 2–3 month old male mice. Tumours were irradiated when they had reached a mean diameter of 8–9 mm. At this size the volume doubling time was about 24 h. The source of radiation was a Pantak x-ray set operated at 240 kV and 15 mA (h.v.l 1·3 mm Cu, dose rate 240 rad/min). Mice were anaesthetized with pentobarbitone sodium before irradiation. Mice which did not receive the sensitizing drug were given 60 mg/kg of the anaesthetic; those receiving the sensitizer had approximately three-quarters of this dose since the sensitizer itself had a mild anaesthetic effect. In some experiments the tumours were irradiated with their blood supply occluded by a semicircular aluminium clamp applied between the tumour and the chest wall 15 min before the start of irradiation, to render all the tumour cells hypoxic (Denekamp and Harris, 1975). Mice treated with Ro-07-0582 were given either 1 mg/g body weight or 0·2 mg/g of the drug (dissolved in saline) by intraperitoneal injection 30 min before the start of irradiation.

In order to measure the gross response of tumours to radiation, each tumour was measured 3–5 times per week over 3 mutually perpendicular diameters until it reached a mean diameter of 13·5 mm, when the mouse was killed. The geometric mean diameter was calculated for each individual tumour for each day. Growth curves were then constructed by plotting the mean diameter for a group of animals receiving the same treatment against time. Dose-effect curves were constructed by measuring the time for each individual tumour to grow from treatment size to 11 mm. Tumours were standardized to a diameter of 8·5 mm at treatment by adding or subtracting a correction throughout for the small difference in size of individual tumours at irradiation. For each radiation dose group a mean time to grow from 8·5 to 11 mm and its 95% confidence limits could then be calculated and plotted as a function of this dose.

For cell survival studies tumours were excised, either immediately or at various times after irradiation, and single cell suspensions prepared as previously described (McNally, 1972). An aliquot of the unirradiated cell suspension was then exposed to a dose of 8 krad of $^{60}$Co gamma rays and $5 \times 10^5$ of these " feeder " cells were mixed with the test cells in 20 ml of Eagle's Minimum Essential Medium plus 15% foetal calf serum and antibiotics plus 0·25% Difco " Noble " agar. 4 ml of this suspension was pipetted into a 50 mm plastic Petri dish containing a 3 ml " base " of 0·9% agar in medium so that the appropriate number of test cells were mixed with $10^5$ " feeder " cells. Four replicate plates were used for each tumour cell suspension. The cells were then incubated for 15–20 days at 37°C in a humidified atmosphere of 5% $CO_2$ in air. Macroscopic colonies were counted and survival curves constructed.

### RESULTS

The times for tumours to grow from treatment size (8·5 mm) to 11 mm diameter after exposure to various doses of x-rays are plotted as a function of the x-ray dose in Fig. 1. The tumours were unclamped and animals were breathing air. The animals treated with Ro-07-0582 had received 1 mg/g 30 min before starting the irradiation. Each point represents data from 6–8 animals, except for that for 2000 rad plus Ro-07-0582 for which there were only 4 mice. The error bars in Fig. 1 represent the 95% confidence limits.

The 2 largest doses of radiation delivered to tumours that had received the sensitizer (2500 and 3000 rad) produced some apparent local cures, in that 2 of the 7 animals whose tumours had

FIG. 1.—Dose effect curves for Sarcoma F. The effect is the time to grow from the diameter when irradiated (8·5 mm) to 11 mm. The vertical lines indicate the 95% confidence limits. The arrows indicate that the points represent minimum estimates of the time to grow to 11 mm. ○ Irradiations in air, ● irradiations in air 30 min after intraperitoneal injection of 1 mg/g Ro-07-0582. The dashed lines represent an extension of the hypoxic component to doses less than 2000 rad and the effect of sensitization of this component by factors 1·4, 2·0 and 2·2 on the overall dose—effect curve.

received 2500 rad and 5 of the 7 that had received 3000 rad had to be killed due to lung metastases at times when there was no evidence of regrowth of the primaries. The 2 points in Fig. 1 indicated by arrows therefore represent minimum estimates of the delay induced by these 2 doses of radiation. The curve for tumours irradiated in the absence of the sensitizer had the biphasic shape characteristic of a mixed population of oxic and hypoxic cells (Thomlinson and

FIG. 2.—Growth curves for tumours irradiated with 1000 rad alone or with 1000 rad plus 1 mg/g Ro-07-0582 immediately after irradiation. The vertical lines indicate 95% confidence limits.

Craddock, 1967). The effect of the sensitizer was to displace the dose at which this biphasic shape became apparent from about 2000 rad to probably well over 2500 rad (Fig. 1).

Even at low doses of radiation some sensitization was apparently produced by Ro-07-0582. However, this was because there was a slight effect of the drug if administered *after* irradiation. Figure 2 shows growth curves for tumours receiving 1000 rad alone or given 1 mg/g of the drug immediately after irradiation. The effect of the drug was to increase the time taken to grow to 11 mm from 5 to 7·3 days. It is not possible to allow for this post-irradiation effect in calculating the radiosensitizing effect of Ro-07-0582 since it has been measured only after one dose of radiation. However, it is not likely to contribute significantly to the measured enhancement ratio since Denekamp and Harris (1975), using a different transplanted tumour in CBA mice (carcinoma NT), showed that for a dose of 2000 rad, if the drug was present before irradiation, the delay in regrowth was 3 times greater than if it was absent but that adding the drug after irradiation only increased the delay by 10–20%.

The enhancement ratio for growth delay measured from Fig. 1 ranges from 1·25 for a growth delay of 9 days to 2·0 for a delay of 15 days. This dependence on the level of damage is because of the biphasic nature of the dose-effect curve for irradiations in the absence of the sensitizer, reflecting the response of a mixed population of oxic and hypoxic cells.

Figure 3 shows survival values for the cells of this tumour irradiated *in vivo* in the absence of the sensitizer and assayed *in vitro*. The animals were breathing air and the tumours were either clamped or unclamped. The line was drawn by eye through the points. Clamping the tumour did not significantly increase the resistance of the cells to radiation, implying a large hypoxic frac-

FIG. 3.—Survival values for the cells of Sarcoma F irradiated *in vivo* and assayed *in vitro*. ○ Animals breathing air, × animals breathing air and the tumours clamped 15 min before irradiation. Typical standard errors are shown on some of the points. The line was drawn by eye through all the points.

tion. The survival curves for cells from unclamped tumours irradiated in the presence of either 1·0 mg/g or 0·2 mg/g of Ro-07-0582 are shown in Fig. 4. The survival curve for cells from unclamped tumours irradiated in the absence of the sensitizer, redrawn from Fig. 3, has been included for comparison. The effect of the sensitizer was essentially dose modifying because of the large hypoxic fraction. The Do for irradiations in the absence of the drug was 350 rad, that for irradiations in the presence of 1 mg/g was 160 rad and for 0·2 mg/g it was 270 rad. The x-ray dose enhancement ratio for each drug concentration can be taken as the ratio of the Do in the absence of the drug to that in its presence because the drug was dose modifying. The enhancement ratio for 1 mg/g Ro-07-0582 was 2·2 and for 0·2 mg/g it was 1·3. This independence of the enhancement ratio on the x-ray dose contrasts with the lack of a significant effect of Ro-07-0582 on growth delay for x-ray doses less than about 1500 rad (Fig. 1).

### DISCUSSION

Figures 1 and 4 clearly demonstrate that Ro-07-0582 is an effective sensitizer of naturally occurring hypoxic cells in tumours. If it can be assumed that the delay in regrowth for doses larger than about 2000 rad is a reflection of the response of hypoxic cells (Fig. 1), then it is possible to estimate an enhancement

FIG. 4.—Survival curves for the cells of Sarcoma F irradiated in air breathing animals in the presence of no drug (○), 0·2 mg/g Ro-07-0582 (△) or 1 mg/g Ro-07-0582 (●).

ratio for hypoxic cells left *in situ*. This was done by constructing hypothetical growth curves assuming uniform sensitization of the hypoxic cells by x-ray dose enhancement ratios of 1·4, 2·0 and 2·2. These hypothetical curves are represented by the dashed lines in Fig. 1. The enhancement ratio of 1·4 does not fit the data. The value of 2·0 probably represents a minimum estimate of the enhancement ratio, while the value of 2·2 is in good agreement with the data and also agrees with that deduced from the survival curves (Fig. 4).

Hewitt (1966) studied the sensitizing effect of high pressure oxygen (HPO) on cell survival in the present tumour system and found no significant sensitization using his dilution assay. He used this tumour because his previous estimate of the hypoxic fraction had indicated a value of about 50% (Hewitt and Wilson, 1961), so that any sensitization by HPO would have been easily detected. Hewitt expressed his results as the ratio of the surviving fraction of cells after irradiation of the tumour in mice breathing air to that after irradiation breathing oxygen, at a given dose of x-rays. Significant sensitization should give a ratio of cell survival greater than 1 by one or more orders of magnitude, particularly at doses greater than 1000 rad. In the Table Hewitt's results are compared with the present ones using Ro-07-0582. The smallest dose he used was 2040 rad but for all the x-ray doses used he found no real effect of breathing oxygen. In the present experiments, however, a dose of 1000 rad killed 25 times more cells in the presence of 1 mg/g Ro-07-0582 than in its absence and a dose of 2040 rad

TABLE.—*The Effects of Ro*-07-0582 *or High Pressure Oxygen on Survival of the Cells of Sarcoma F Assayed either* in vitro (*Present Results*) *or* in vivo (*Hewitt*, 1966) *after Irradiation* in vivo

| Dose (rad) | Surviving fraction (air)* / Surviving fraction Ro-07-0582 (present results) | Surviving fraction (air)* / Surviving fraction (HPO) (Hewitt, 1966) |
|---|---|---|
| 1000 | 25 | — |
| 2040 | >100 | 4·8 |
| 2260 | — | 0·73 |
| 2580 | — | 2·8 |
| 3280 | — | 1·7 |

HPO 45 p.s.i.

would, by extrapolation of the curves of Fig. 4, increase this ratio to over 100. The absence of an effect of oxygen in Hewitt's experiment may have been because of its rapid metabolism, or because of a vasoconstrictive effect of the high pressure (*e.g.* Lambertsen, 1966).

The 2 methods of assay used in the present study gave quite different estimates of the hypoxic fraction of cells in the tumour even though they gave the same estimate of the enhancement ratio for Ro-07-0582. As in Hewitt's results, the hypoxic fraction was apparently well over 50% when it was determined by the assay of cells *in vitro* since (*a*) there was little effect of clamping the tumour on cell survival (Fig. 3) and (b) Ro-07-0582 was essentially dose modifying (Fig. 4). In contrast, the growth delay curves (Fig. 1) suggest that the " effective " *in situ* hypoxic fraction was probably well below 10% because (a) the resistant portion did not affect the growth delay curves at doses less than about 2000 rad and (b) there was little effect of Ro-07-0582 below this dose.

Two other estimates of the hypoxic fraction in sarcoma F have been made. Hewitt and Wilson (1961), as mentioned above, deduced that the hypoxic fraction was about 50% when they assayed the cells by their dilution method in which cells are irradiated *in vivo*, removed and subsequently injected into other mice. Begg (personal communication) deduced that there was a relatively low hypoxic fraction using an assay which, like growth delay, does not involve removal of cells from their normal environment. He measured the amount of radioactivity in tumours as a function of time after a single intraperitoneal injection of $^{125}$I-iododeoxyuridine to the mice. This technique can be used to assess quantitatively the death of cells *in vivo* following irradiation (Hofer, 1970; Begg and Fowler, 1974). Begg found a large difference in the doses of x-rays needed to produce the same loss of radioactivity when tumours were irradiated either clamped or unclamped, indicating that in the unclamped situation the proportion of hypoxic cells was small.

Thus, the 2 techniques in which cells are left *in situ* (growth delay and loss of $^{125}$I activity) gave lower estimates of the hypoxic fraction than those in which the cells are removed from the mice after irradiation (cell survival in Petri dishes and in recipient mice). A possible explanation for this discrepancy is that " doomed " hypoxic cells which would die if left *in situ*, even though they have survived the radiation, are " rescued " from death due to hypoxia when the tumour is excised and a single cell suspention obtained (McNally, 1973).

In order to test this possibility, unclamped tumours were exposed to single doses of 2000 rad (animals breathing air), excised at various times after irradiation and the cells assayed for their colony forming ability. If hypoxic cells that had survived the irradiation died due to hypoxia or other nutrient deficiency, there should be a fall in survival as the interval between irradiation and excision increased. Figure 5 shows that this was not the case; the surviving fraction of cells increased with time up to 8 h by a factor of 5–10 and showed no consistent change thereafter. The scatter in the data does not exclude a small fall in

FIG. 5.—The effect of time between irradiation *in vivo* and excision on the survival *in vitro* of the cells of Sarcoma F exposed to a single dose of 2000 rad.

survival, within the first hour or two, but this is certainly not sufficient to account for the difference in the estimates of the hypoxic fraction. The observed increase in surviving fraction with time (Fig. 5) is probably due to recovery from potentially lethal damage (Hahn and Little, 1972), although if cells which had been killed by the radiation become pycnotic and lysed soon after irradiation so that they were not counted in the haemacytometer, this could lead to an apparent increase in survival. This possibility cannot be excluded although the yield of cells by the trypsinization procedure and the proportion of intact cells in the haemacytometer did not noticeably change with time between irradiation and excision of the tumour. It can be concluded that either there was no progressive decrease in cell survival due to hypoxia, or the recovery from potentially lethal damage more than compensated for such hypoxic death.

Brown and Howes (1974) have proposed another explanation for the discrepancy in the estimates of the hypoxic fraction. They suggested that vascular damage can contribute to the observed growth delay, causing extra delay in regrowth of tumours in air-breathing animals. The effect of this would be to displace the transition region from an oxic to a hypoxic response to larger doses of radiation than if such damage were absent, thus decreasing the estimate of the hypoxic fraction. Reasons why such an explanation may not apply have been discussed previously (McNally, 1974) and will not be repeated here. Nevertheless, we must consider the consequences of applying this explanation to the present results. If the effective hypoxic fraction *in situ* were in excess of 50% as the survival curves imply (Fig. 3), this would mean that even at the lowest dose of radiation used in the absence of the sensitizer (1000 rad) the growth delay should reflect primarily the response of hypoxic cells plus this vascular damage. One would not then expect to see a biphasic response such as in Fig. 1 unless a portion of the vascular endothelium were hypoxic. This seems unlikely. Further, there should be an effect of Ro-07-0582 at doses less than 2000 rad unless the effect of the vascular damage far outweighed that of cell killing in causing growth delay. This, too, seems unlikely.

A more likely explanation for the discrepancy in the estimates of the hypoxic fraction is that assays which involve removal of cells from their normal environment after irradiation do not accurately reflect the course of events in the undisturbed tumour (McNally, 1973). It is known that the degree of intercellular contact can affect a cell's ability to absorb radiation damage as sub-lethal (Durand and Sutherland, 1972). In tumours, it may differentially affect the radioresistance of hypoxic and aerobic cells. In particular, hypoxic cells may be more radiosensitive when left *in situ* than when plated *in vitro*. Alternatively, the present results are consistent with more radioresistance of cells in contact (*i.e. in situ*) than when separated, if the cells are aerobic but not if they are hypoxic.

In summary, the results presented in this paper demonstrate that the 2-nitroimidazole Ro-07-0582 is an effective sensitizer of naturally occurring hypoxic tumour cells in a system where high pressure oxygen has been shown to have no effect. The sensitizing enhancement ratio deduced from the growth curves in which cells are left *in situ* (Fig. 1) is in agreement with that measured by the *in vitro* assay of cell survival (Fig. 4). However, the 2 methods of assay give quite different estimates of the hypoxic fraction. This discrepancy is probably a reflection of differences in the expression of radiation damage by cells left *in situ* and those assayed *in vitro*.

I thank Dr J. F. Fowler for helpful discussions, Mrs J. de Ronde for her expert technical assistance and Misses A. Walder, A. Marriott and J. Radmore for provision and care of the animals.

### REFERENCES

ASQUITH, J. C., WATTS, M. E., PATEL, K., SMITHEN, C. E. & ADAMS, G. E. (1974) Electron-affinic Sensitisation V. Radiosensitisation of Hypoxic Bacteria and Mammalian Cells *in vitro* by some Nitroimidazoles and Nitropyrazoles. *Radiat. Res.*, **60**, 108.

BEGG, A. C. & FOWLER, J. F. (1974) A Rapid Method for the Determination of Tumour RBE. *Br. J. Radiol.*, **47**, 154.

BREUR, K. (1966) Growth Rate and Radiosensitivity of Human Tumours. I. Growth Rate of Human Tumours. *Eur. J. Cancer*, **2**, 157.

BROWN, J. M. & HOWES, A. E. (1974) Comparison of Tumour Growth Delay with Cell Survival. *Br. J. Radiol.*, **47**, 509.

DENEKAMP, J. & HARRIS, S. R. (1975) Tests of Two Electron-affinic Radiosensitisers *in vivo* using Regrowth of an Experimental Carcinoma. *Radiat. Res.*, **61**, 191.

DURAND, R. E. & SUTHERLAND, R. M. (1972) Effects of Intercellular Contact on Repair of Radiation Damage. *Expl cell Res.*, **71**, 75.

FOWLER, J. F. & ADAMS, G. E. (1975) Radiosensitisation of Hypoxic Cells in Solid Tumours in Mice. *Br. J. Radiol.*, **48**, 77.

HAHN, G. M. & LITTLE, J. B. (1972) Plateau-phase Cultures of Mammalian Cells. *Current Top. Radiat. Res. Quart.*, **8**, 39.

HEWITT, H. B. (1966) The Effect on Cell Survival of Inhalation of Oxygen under High Pressure during Irradiation *in vivo* of a Solid Mouse Sarcoma. *Br. J. Radiol.*, **39**, 19.

HEWITT, H. B. & WILSON, C. E. (1961) Survival Curves for Tumor Cells Irradiated *in vivo*. *Ann. N.Y. Acad. Sci.*, **95**, 818.

HOFER, K. G. (1970) Radiation Effects on Death and Migration of Tumor Cells in Mice. *Radiat. Res.*, **43**, 663.

LAMBERTSEN, C. J. (1966) Physiological Effects of Oxygen Inhalation at High Partial Pressures. In *Fundamentals of Hyperbaric Medicine*. Pub. No. 1298. Washington, D.C.: National Academy of Sciences.

McNALLY, N. J. (1972) Recovery from Sublethal Damage by Hypoxic Tumour Cells *in vivo*. *Br. J. Radiol.*, **45**, 116.

McNALLY, N. J. (1973) A Comparison of the Effects of Radiation on Tumour Growth Delay and Cell Survival. The Effect of Oxygen. *Br. J. Radiol.*, **46**, 450.

McNALLY, N. J. (1974) Tumour Growth Delay and Cell Survival *in situ*. *Br. J. Radiol.*, **47**, 510.

McNALLY, N. J. (1975) A Comparison of the Effects of Radiation on Tumour Growth Delay and Cell Survival. The Effect of Radiation Quality. *Br. J. Radiol.*, **48**, 141.

THOMLINSON, R. H. & CRADDOCK, E. A. (1967) The Gross Response of an Experimental Tumour to Single Doses of X-rays. *Br. J. Cancer*, **21**, 108.